# Health outcomes of maternal smoking during pregnancy and postpartum period for the mother and infant: protocol for an umbrella review

**DOI:** 10.1186/s13643-018-0900-9

**Published:** 2018-12-19

**Authors:** Tuba Saygın Avşar, Hugh McLeod, Louise Jackson

**Affiliations:** Health Economics Unit, Institute of Applied Health Research, College of Medical and Dental Sciences, University of Birmingham, Edgbaston, Birmingham, B15 2TT UK

**Keywords:** Smoking during pregnancy, Health outcomes, Overview of reviews, Umbrella review, Systematic review

## Abstract

**Background:**

Internationally, tobacco smoking is a leading cause of mortality, morbidity and health inequality. In England, despite increasing awareness about importance of public health interventions to reduce smoking, about 10% of pregnant women are known to be smokers at the time of delivery. There are many systematic reviews investigating the impact of maternal smoking during pregnancy on particular health conditions. Hence, this overview of systematic reviews, which aims to include all health conditions for mother and infant caused by smoking during pregnancy, is timely.

**Methods:**

CINAHL, EMBASE, MEDLINE, PsycINFO, Web of Science, CRD Database (includes DARE, NHSEED and HTA) and HMIC databases will be searched for systematic reviews investigating the effects of smoking during pregnancy. Only reviews written in English and published by 31/12/17 will be included. Studies focussed on low-income countries will be excluded. Study selection and quality assessment will be completed by two reviewers independently. To assess the quality of included studies, the Centre for Reviews and Dissemination checklist for systematic reviews will be utilised.

**Discussion:**

Existing systematic reviews focus on the impact of smoking during pregnancy on a specific health condition. This review aims to analyse current evidence on the overall health outcomes associated with smoking whilst pregnant by providing an overview of evidence from systematic reviews.

**Systematic review registration:**

PROSPERO CRD42018086350.

**Electronic supplementary material:**

The online version of this article (10.1186/s13643-018-0900-9) contains supplementary material, which is available to authorized users.

## Introduction

### Background

Smoking is the highest preventable cause of numerous health problems worldwide [1]. Seven million people die every year because of smoking in the world and more than 18% of adults smoke daily in OECD countries [2]. In England, 16% of all deaths were attributed to smoking in 2015 [3]. Smoking during pregnancy is responsible for many avoidable health conditions and deaths across the countries [4]. Smoking status at delivery was 10.5% in the UK in 2016/2017, and the estimated annual cost to the NHS was up to £87 million in 2010 [5, 6].

Smoking is the leading cause of inequalities in health across and within countries [7], and there is a negative correlation between education and income levels and smoking [8]. In line with this, the smoking status of pregnant women at the time of delivery is higher in deprived areas of England, being 27% in Blackpool compared to 2% in Central London [5].

There are many systematic reviews investigating the impact of maternal smoking during pregnancy on individual health conditions, but there have been few studies seeking to review evidence across the range of health conditions caused by maternal smoking during pregnancy. In 2010, Godfrey et al. [6] reported a scoping review of the health outcomes associated with smoking during pregnancy. However, their strategy focused on search terms for a limited number of smoking-related health conditions which meant that the review may not have captured some relevant health conditions. In addition, quality assessment of the included studies and reviews was not conducted. Several narrative reviews have surveyed short- and long-term effects of maternal smoking during pregnancy and lactation and presented evidence around the topic [9, 10]. Nevertheless, these reviews did not systematically assess all available evidence, instead mostly focussed on the negative health effects of nicotine exposure reported in some studies. Considering the large number of published systematic reviews of observational studies regarding the impact of maternal smoking during pregnancy on different health outcomes, an overall evaluation of the current evidence is timely.

### Objective of the review

This review seeks to investigate the impact of smoking during pregnancy and the postpartum period on health outcomes for the mother and infant in developed country settings to inform future research and health policy.

## Methods/design

This umbrella review is designed in line with the objectives and guideline provided by Cochrane Handbook for Systematic Reviews of Interventions [11].

### Inclusion criteria

Studies will be included based on the following eligibility criteria.

#### Population

Smoking behaviour, tobacco regulations, access to care for pregnant women, and other related factors are different in high- and middle-income countries compared to low-income countries, and consequently, health outcomes of smoking during pregnancy may not be the same [12–17]. For this reason, systematic reviews of studies focussing on low-income countries will be excluded [18]. There will not be any exclusions based on age or social groups.

#### Intervention/effect

This review will focus on the health impacts of maternal smoking during pregnancy and the postpartum period. Therefore, studies that investigated the effect of maternal smoking during pregnancy and postpartum period will be included.

#### Comparator

The comparator is defined as pregnant or postpartum women who have never smoked or who have quit smoking.

#### Outcome measure

The primary outcome measures for this review are the health outcomes of smoking during pregnancy and the postpartum period for the mother and infant. Outcomes include pregnancy-related clinical problems and long-term adverse health outcomes for the infant. Measures may include odds ratios and relative risks for smoking women and their children compared to non-smoking women and their children.

#### Study design

Only systematic reviews published in a peer-reviewed journal will be included in the review.

#### Language

For pragmatic reasons, this review will only include systematic reviews written in English.

#### Publication date

This study will include systematic reviews published up to 31 December 2017.

### Search strategy

A scoping search was conducted using MEDLINE with the words “pregnant women”, “pregnant smokers”, “maternal smoking”, “health outcomes”, “cost outcomes”, and “QALYs”. Then, the InterTASC Information Specialists Sub-Groups (ISSG) filter was used to identify keywords. Additionally, the keywords of five systematic reviews in relevant topics were reviewed. Identified keywords were discussed with two experts to crosscheck. The chosen search terms are shown in Additional file 1: Appendix 1.

The literature search strategy of this review is defined as follows:

1. The selected keywords within each concept will be combined with “OR”, and concepts will be combined with “AND”.
2. The inclusion criteria will be piloted by the reviewers independently in order to maximise the consistency of the study selection process.
3. CINAHL, EMBASE, MEDLINE, PsycINFO, Web of Science, CRD Database (includes DARE, NHSEED and HTA) and HMIC databases will be searched with those identified keywords. In addition, references of selected studies will be searched for relevant articles.
4. The phase of screening for eligibility will be conducted by two reviewers independently. Any discrepancies will be resolved by discussion with a third reviewer.

### Data extraction

A data extraction sheet was created which covers the lead author’s name and publication year, study design and the databases searched, number of studies included, main outcomes, and some other methodological information (Additional file 1: Appendix 2). The extraction tool will be piloted. One reviewer (TS) will extract the data and another reviewer (HM) will check the extracted data to minimise any bias.

### Data management

Data management will be done by using ENDNOTE and Microsoft Excel software.

### Quality assessment

The Centre for Reviews and Dissemination’s practical checklist for conducting a critical appraisal of systematic reviews was modified according to the needs of the current study [19]. For instance, questions on protocol, publication bias and heterogeneity were added (Additional file 1: Appendix 3). Quality assessment will be done by two reviewers independently. Any discrepancies will be solved through discussion or involvement of a third reviewer.

### Analysis and presentation of the results

The study selection process will be summarised by using a PRISMA diagram [20]. A narrative analysis of the data gathered via the systematic review will be undertaken. As the study will include reviews focussing on varied health conditions, no sub-group analysis has been planned. Results will be presented in accordance with the Cochrane Handbook for Systematic Reviews of Interventions guidelines [11].

## Discussion

Existing systematic reviews focus on the impact of smoking during pregnancy on particular health conditions. This review aims to draw a broader picture of the current evidence by including systematic reviews that investigated any health outcome associated with smoking whilst pregnant.

## Additional file

Additional file 1: Appendix 1: Sample search strategy from MEDLINE. Appendix 2: Data extraction tool. Appendix 3: Critical appraisal checklist for systematic reviews. (DOCX 23 kb)

## Abbreviations

CRD: Centre for Reviews and Dissemination
DARE: Database of Abstracts of Reviews of Effects
HMIC: The Healthcare Management Information Consortium
HTA: Health Technology Assessment
NHSEED: NHS Economic Evaluation Database
PRISMA: Preferred Reporting Items for Systematic Review and Meta-Analysis
