# Supplementary material for: Health outcomes of maternal smoking during pregnancy and postpartum period for the mother and infant: protocol for an umbrella review
Source: Syst Rev. 2018 Dec 19;7:235. doi: 10.1186/s13643-018-0900-9 (PMC6299640; doi:10.1186/s13643-018-0900-9)
Supplement: Supplementary file 1 — Appendix 1: Sample search strategy from MEDLINE. Appendix 2: Data extraction tool. Appendix 3: Critical appraisal checklist for systematic reviews. (DOCX 23 kb) [file 13643_2018_900_MOESM1_ESM.docx]

**Appendix 1: Sample Search Strategy from MEDLINE**

1. pregnan*.mp.
2. exp Pregnancy/
3. postpartum.mp. or exp Postpartum Period/
4. 1 or 2 or 3
5. exp Smoking/ or smok*.mp.
6. exp Tobacco/ or tobacco.mp.
7. 5 or 6
8. 4 and 7
9. pregnancy outcomes.mp. or exp Pregnancy Outcome/
10. birth outcomes.mp. or exp Infant, Newborn/
11 . exp Child Development/ or childhood outcomes.mp.
12. exp Risk Factors/ or adverse outcomes.mp.
13. "quality of life".mp. or exp "Quality of Life"/
14. "long term outcomes".mp. or exp Follow-Up Studies/
15. 9 or 1 0 or 11 or 12 or 13 or 14
16. 8 and 15
17. systematic review.mp. or exp "systematic review"/
18. meta-analysis.mp. or exp Meta-Analysis/
19. 18 or 19
20. 16 and 19
21 . limit 20 to (english language and humans)

**Appendix 2: Data Extraction Tool**

| Date |  |
| --- | --- |
| First Author & Publication Year |  |
| Study Focus |  |
| Mother or Infant Investigated (M/I) |  |
| Meta-analysis (Y/N) |  |
| Years Included |  |
| Databases Searched |  |
| Any Limitations to Database Search |  |
| Setting/Country of Included Studies |  |
| Number of Included Studies |  |
| Main Outcomes |  |
| Heterogeneity |  |
| Quality Assessment Tool |  |
| Dose-response Test |  |
| Partner Inclusion |  |
| Number of Exposed |  |
| Number of Unexposed |  |
| Number of Affected |  |
| Number of Not Affected |  |
| First Author & Publication Year |  |
| Study Focus |  |
| Years Included |  |
| Databases Searched |  |
| Any Limitations to Database Search |  |
| Setting/Country of Included Studies |  |
| Number of Included Studies |  |
| Main Outcomes |  |
| Heterogeneity |  |
| Quality Assessment Tool |  |
| Dose-response Test |  |
| Partner Included |  |

**Appendix 3: Critical Appraisal Checklist for Systematic Reviews**

| *No* | *Statement* | *YES/NO/ NA/*  *UNCLEAR* |
| --- | --- | --- |
| 1 | *Was a protocol prepared prior to the review?^[[1]](#footnote-1)^* |  |
| 2 | Was the review question clearly defined in terms of population, interventions (phenomena of interest), comparators, outcomes and study designs (PICOS)? |  |
| 3 | Was the search strategy adequate and appropriate? |  |
| 4 | Were there any restrictions on language, publication status or publication dates? |  |
| 5 | Were preventative steps taken to minimise bias and errors in the study selection process? |  |
| 6 | Was quality assessment done? |  |
| 7 | Were preventative steps taken to minimise bias and errors in the quality assessment process? |  |
| 8 | Were preventative steps taken to minimise bias and errors in the data extraction process? |  |
| 9 | Were adequate details presented for each of the primary studies? |  |
| 10 | Were appropriate methods used for data synthesis? |  |
| 11 | Were differences between studies assessed? |  |
| 12 | Were the studies pooled, and if so was it appropriate and meaningful to do so? |  |
| 13 | *Was heterogeneity tested?* |  |
| 14 | *Were the causes of heterogeneity assessed?* |  |
| 15 | *Was publication bias assessed?* |  |
| 16 | Do the authors’ conclusions accurately reflect the evidence that was reviewed? |  |

**Reviewer: Date: Author & Year: Record Number:**

1. Italic ones were added by the authors. [↑](#footnote-ref-1)
